# Age‐related hearing loss and dementia‐related neuropathology: An analysis of the United Kingdom brains for dementia research cohort

**DOI:** 10.1111/bpa.13188

**Published:** 2023-08-08

**Authors:** Jessica A. Katanga, Calum A. Hamilton, Lauren Walker, Johannes Attems, Alan J. Thomas

**Affiliations:** ^1^ Translational and Clinical Research Institute Newcastle University Newcastle upon Tyne UK

**Keywords:** cognitive, dementia, hearing, impairment, loss, neuropathology

## Abstract

Age‐related hearing loss frequently precedes or coexists with mild cognitive impairment and dementia. The role specific neuropathologies play in this association, as either a cause or a consequence, is unclear. We therefore aimed to investigate whether specific dementia related neuropathologies were associated with hearing impairment in later life. We analysed data on ante‐mortem hearing impairment with post‐mortem neuropathological data for 442 participants from the Brains for Dementia Research Cohort. Binary logistic regression models were used to estimate the association of hearing impairment with the presence of each dementia‐related neuropathology overall, and with specific staged changes. All analyses adjusted for age and sex, and several sensitivity analyses were conducted to test the robustness of findings. Presence and density of neuritic plaques were associated with higher odds of hearing impairment ante‐mortem (OR = 3.65, 95% CI 1.78–7.46 for frequent density of plaques). Presence of any LB disease was likewise associated with hearing impairment (OR = 2.10, 95% CI 1.27–3.48), but this did not increase with higher cortical pathology (OR = 1.53, 95% CI 0.75–3.11). Nonspecific amyloid deposition, neurofibrillary tangle staging, overall AD neuropathology level, and cerebrovascular disease were not clearly associated with increased risks of hearing impairment. Our results provide some support for an association between dementia‐related neuropathology and hearing loss and suggest that hearing loss may be associated with a high neuritic plaque burden and more common in Lewy body disease.

## Graphical abstract

Photomicrographs taken from the temporal cortex. Case 20174966 exhibits severe tau pathology as neurofibrillary tangles (a ‐ arrow) and frequent neuritic plaques (a ‐ arrowhead), abundant amyloid beta plaques (b ‐ arrows) and severe cerebral amyloid angiopathy (b ‐ arrowhead. Scale bar in (a) is 100 μm and is valid for images a and b.

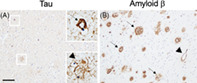

## 1 INTRODUCTION

Age‐related hearing loss (ARHL) frequently precedes or coexists with mild cognitive impairment (MCI) and dementia [1, 2], and the presence of hearing impairment is associated with greater risk of developing dementia in cognitively healthy people [3]. However, the causal direction of this association, and role of underlying neuropathological changes, is unclear.

ARHL may lead to subsequent cognitive impairment through ‘sensory deprivation’ as it has been suggested that, over time, the constant diversion of neural energy needed to compensate for hearing loss leads to permanent neurological changes that disadvantage higher processing networks [4]. Evidence to support this includes the observations of Lin et al. [5], who showed hearing loss is associated with accelerated brain atrophy within right superior, middle and inferior temporal gyri: loci for language processing, semantic memory and multimodal sensory integration [6]. These changes could contribute to neuronal vulnerability for neuropathological change and/or lower the threshold needed for neuropathological changes to manifest in cognitive symptoms [7].

A factor also worth considering in terms of what may cause hearing loss to lead to cognitive impairment is social withdrawal. In a cross‐sectional survey of 3613 older adults (age >50), hearing impairment was an independent, positive correlate for isolation and loneliness [8]. Loneliness has been shown by meta‐analysis to positively correlate with developing dementia—RR 1.26 (95% CI 1.14–1.40) [9] and isolation, defined by having fewer social interactions and a smaller social network [10] predicts sharper decline in the cognitive function of older adults [11, 12].

Conversely, hearing impairment may be a consequence of neurodegeneration‐related cognitive impairment. The ‘cognitive ear’—the interface between auditory and cognitive processing—[13, 14] enables healthy individuals to process, select, distinguish, and understand an array of sounds amidst a complex soundscape [15]. Dementia‐related structural and functional brain changes may therefore have knock‐on effects on auditory processing: neurodegenerative pathology may involve the auditory cortex [16], for example, neurofibrillary tangle (NFT) Braak stage VI (i.e., highest NFT Braak stage) includes the involvement of the auditory neocortex [17]. ARHL preceding dementia is not incompatible with this notion, as older adults with MCI have been shown to have more latent and less frequent auditory event related potentials than those without MCI, suggesting that changes in auditory neural transmission may occur early in the disease course [18].

To better understand the routes through which ARHL is associated with cognitive dysfunction the relationship between ARHL and dementia‐related neuropathological changes—including Alzheimer's‐related amyloid and tau pathology, Lewy body pathology, and cerebrovascular disease, have to be clarified.

Previous research examining the associations between ARHL, and suspected Alzheimer's pathology have applied PET imaging of amyloid and tau in individuals with ARHL– [19, 20, 21, 22]. Three studies found an increased burden of amyloid and tau in those with ARHL, supporting a link between ARHL and Alzheimer's disease related brain changes. However, one study found no such association [22].

Whilst PET imaging is superior to clinical evaluation alone for the detection of Alzheimer or Lewy pathology [23] it is still an indirect measure and post‐mortem neuropathological assessment remains the gold standard.

Two studies interrogated the association between hearing impairment and post‐mortem neuropathological data. The largest comprises of 2755 individuals from the National Alzheimer's Coordinating Centre (NACC) database. Impaired hearing was associated with higher Braak NFT stage in cognitively healthy older adults, higher neocortical LB presence in MCI, and micro infarcts in dementia, but negatively correlated with neuritic plaque density in the latter [24]. The other, smaller study of 273 individuals, however found no clear differences in pathological burden of those with hearing impairment compared to those without [25].

The aim of our study, therefore, was to further elucidate whether the presence of dementia related neuropathology is associated with hearing impairment in later life. We have used clinical and post‐mortem neuropathological data from the Brains for Dementia Research (BDR) programme.

## 2 MATERIALS AND METHODS

### 2.1 Participants

Participants were identified from the United Kingdom BDR cohort, which recruited from six regional centres: Oxford, Bristol, Newcastle, Cardiff, London and Manchester. Four hundred and forty‐two participants were eligible for inclusion who had hearing function reported ante‐mortem, and comprehensive neuropathological assessment post‐mortem.

Participants provided written, informed consent prior to assessment and eventual brain tissue donation.

### 2.2 Clinical assessment

During life, participants in the BDR cohort underwent repeated clinical assessment, approximately annually. These clinical assessments gather data on ethnicity, comorbidities, any sensory impairment, smoking, socioeconomic status, education level and cognitive/dementia status—and were conducted by research psychologists or research nurses. Where available, assessments included informants (friends or family members) to provide additional data.

Participants were classified as having dementia, having MCI or being cognitively normal in accordance with methods outlined in McAleese et al paper [26]. This classification utilises information collated from medical records, questionnaires to participant's informant, and neuropsychological testing, specifically the Mini Mental State Exam (scores 27–30 cognitively normal, 24–27 MCI and ≤23 dementia) [26].

### 2.3 Hearing assessment

Participants' hearing status was rated by the researcher at interview, incorporating information from the participant and any informants, as well as their own observations:

0—Normal hearing, no hearing aid used;

1—Hears normally with a hearing aid;

2—Slightly impaired hearing even with a hearing aid;

3—Very impaired hearing even with a hearing aid;

4—Impaired hearing, but no hearing aid used.

For our analyses, those with a rating of 0 were classified as having unimpaired hearing (*n* = 279) and all other ratings as evidence of impaired hearing (*n* = 153).

### 2.4 Neuropathological assessment

All centres adhered to standardised, consensus‐based guidelines for the identification and reporting of neuropathological findings, which included Thal phase of amyloid‐β deposition [27], NFT Braak staging of NFTs [17], Consortium to Establish a Registry for Alzheimer's Disease (CERAD) score of neuritic plaque density [28]. These separate classifiers were then combined in accordance with the NIA‐AA criteria as ‘none’, ‘low’, ‘intermediate’ or ‘high’ level of AD neuropathological change [29]. In addition, Lewy body pathology was assessed using Braak LB staging [30] and McKeith classification [31]. Due to limitations inherent to both systems, with high non‐classifiability [32], information from both sources were harmonised to distinguish cases with any LB pathology according to either system (Braak LB stage ≥1, or any McKeith classification), and in sensitivity analyses constrained to those with a high degree of LB pathology (Braak LB stage ≥4 or limbic/neocortical McKeith classification) or neocortical LB pathology (Braak stage 6 or neocortical McKeith classification) alone. Cerebrovascular pathology was assessed according to the Vascular Cognitive Impairment Neuropathology Guidelines (VCING) criteria [33]: namely, presence of one or more subcortical cerebral infarcts >10 mm, presence of moderate or severe occipital leptomeningeal cerebral amyloid angiopathy, and presence of moderate or severe occipital white matter arteriolosclerosis.

### 2.5 Analysis

Binary logistic regression models were used to estimate the association of hearing impairment with the presence of each dementia‐related neuropathology overall, and with specific staged changes. All analyses were conducted in SPSS statistical software, adjusting for age and sex; pathologies were included as categorical variables predictors, with the reference being the absence or lowest stage of each pathology and significance set at *p* < 0.05.

We conducted sensitivity analyses to test the robustness of these findings, stratifying the cohort by antemortem cognitive status, examining specific subcomponents of Alzheimer's pathology rating (specifically, NIA‐AA ABC staging), and different severity of Lewy body pathology (any LB pathology, high LB pathology, or neocortical LB pathology).

## 3 RESULTS

Baseline characteristics of the cohort are presented in Table 1. Of the 432 participants, 64.6% had normal hearing, and 35.4% had impaired hearing.

**TABLE 1:** Baseline characteristics of sample, stratified by hearing status.

	Normal hearing (*n* = 279)	Impaired hearing (*n* = 153)
Female sex	127 (45.5%)	74 (48.4%)
Age at visit	78.4 (8.9)	84.6 (8.0)
Age at death	81.6 (9.1)	88.1 (7.9)
Cognitively impaired (MCI or Dementia)	179 (64.2%)	79 (51.6%)
Education	12.8 (6.1)	16.1 (4.7)
Use hearing aid	n/a	108 (70.6%)

*Note*: Count (%) and Mean (SD).

### 3.1 Alzheimer's disease neuropathology

**FIGURE 1:**
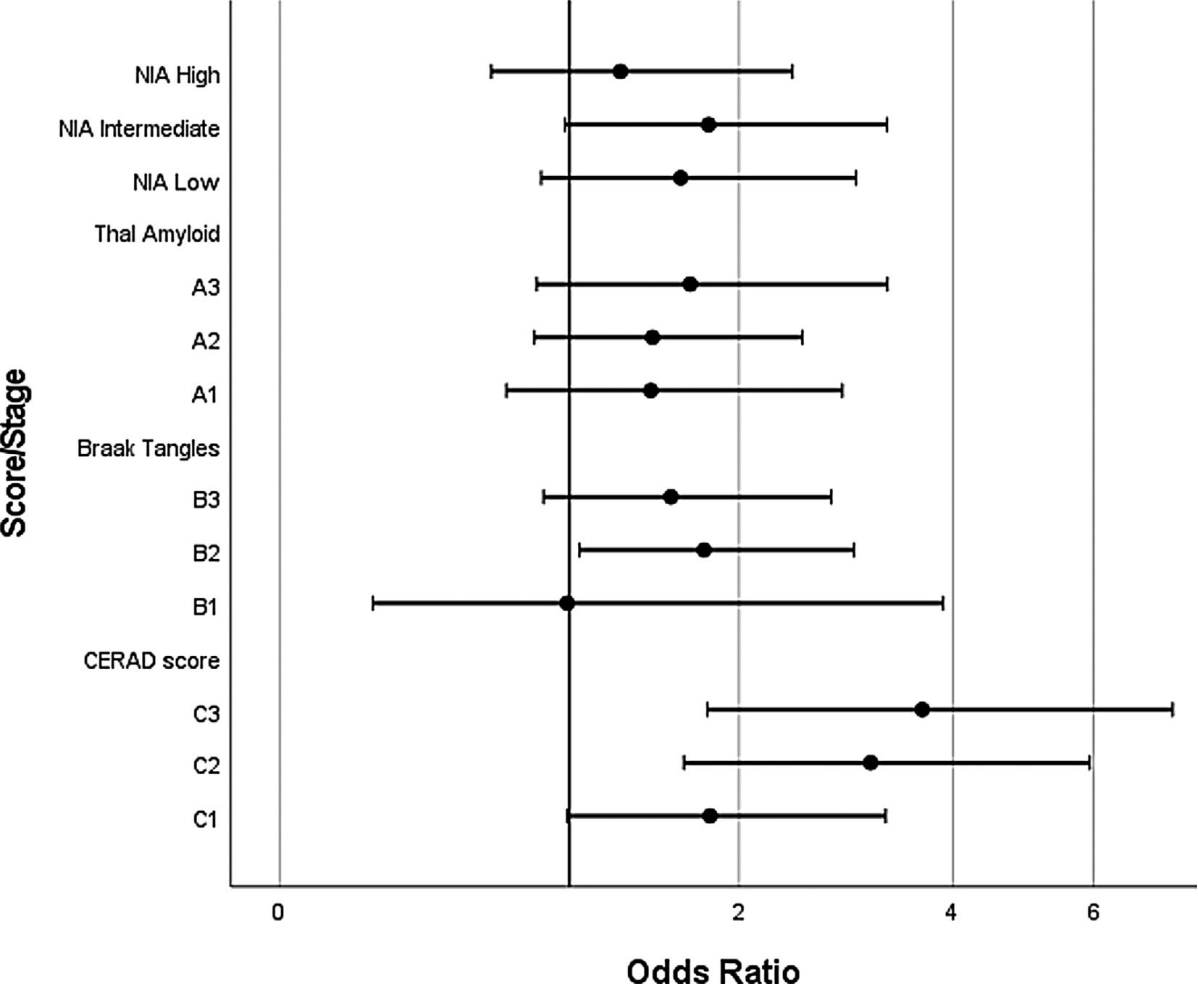
Dot and whisker plot to show associations of Alzheimer's disease pathology with hearing impairment.

Presence of moderate neuritic plaques (C2) and frequent neuritic plaques (C3) both had a significant association with hearing impairment–(OR = 3.11, 95% CI = 1.63–5.94) and (OR = 3.65, 95% CI = 1.78–7.46), respectively. However, for sparse neuritic plaques (C1), the result was non‐significant (OR = 1.80, 95% CI = 0.99–3.26; see Figure 1).

NIA‐AA low (OR = 1.61, 95% CI = 0.87–2.97), intermediate (OR = 1.79, 95% CI = 0.98–3.27) and high (OR = 1.26, 95% CI = 0.66–2.41) levels of AD‐related neuropathological change were not associated with significantly increased odds of hearing impairment in comparison to those without AD‐related neuropathology.

There was no significant association between increasing amyloid deposition phase (A1–A3) and presence of hearing impairment (see Figure 1).

Any association between low levels of NFT pathology (B1) and hearing impairment was highly uncertain (OR = 0.99, 95% CI = 0.25–3.89); moderate tangle pathology (B2) was associated with significantly higher odds of hearing impairment (OR = 1.76, 95% CI = 1.05–2.95), but this was not consistent to the highest stages of tangle pathology (B3), where the association was non‐significant (OR = 1.55, 95% CI = 0.88–2.74).

### 3.2 Lewy body pathology

**FIGURE 2:**
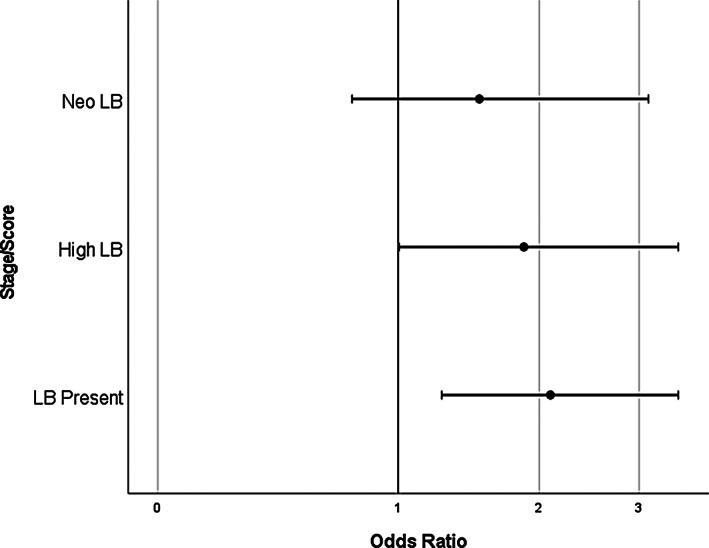
Dot and whisker plot to show associations of Lewy body pathology with hearing impairment.

The presence of any LB pathology was associated with significantly increased likelihood of hearing impairment (OR = 2.10, 95% CI = 1.27–3.48). In sensitivity analyses, this effect remained, albeit attenuated and marginally significant, when restricted to cases with a high degree of LB pathology only (OR = 1.87, 95% CI = 1.01–3.48), but was not significant if restricted to those with neocortical LB pathology only (OR = 1.53, 95% CI = 0.75–3.11; see Figure 2).

When examining specific Braak LB staging, the data were consistent with the presence of any LB pathology being associated with increased risk of hearing impairment, but not with an increasing risk with increasingly higher stage pathology.

### 3.3 Cerebrovascular pathology

**FIGURE 3:**
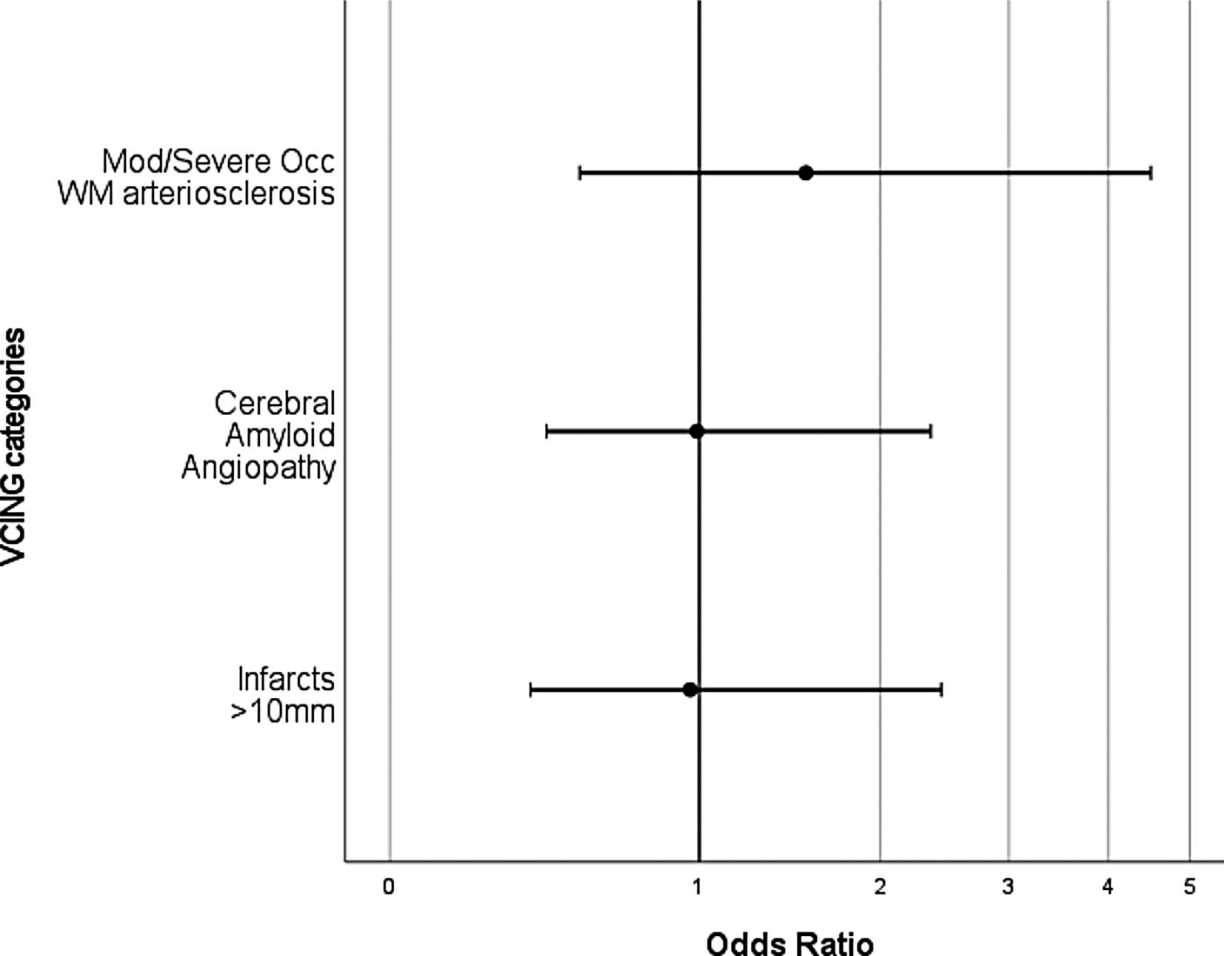
Dot and whisker plot to show odds of hearing impairment associated with each vascular pathology stage/score.

There were no statistically significant associations between any aspects of cerebrovascular disease and presence of hearing impairment (see Figure 3).

Infarcts >10 mm (VCING 1) OR was 0.96, 95% CI 0.37–2.44, Cerebral amyloid angiopathy (VCING 2) OR was 0.99, 95% CI 0.42–2.36 and WM arteriosclerosis (VCING 3) OR was 1.54, 95% CI 0.53–4.50 (see Figure 3). A sensitivity analysis was conducted assessing overall VCING classification (low/moderate/high), which likewise indicated that there were no significant associations between overall cerebrovascular pathology and presence of hearing impairment.

### 3.4 Sensitivity analysis

In a sensitivity analysis, restricting the data to only those with cognitive impairments provided results broadly similar to the primary analyses: moderate and frequent densities of neuritic plaques at autopsy were associated with significantly increased odds of hearing impairment ante‐mortem, whilst Thal amyloid phase and Braak staging were not significantly associated with hearing impairment. Presence of any LB pathology, or high LB pathology specifically, were likewise associated with hearing impairment. Cerebrovascular disease in cognitively impaired individuals was not significantly predictive of hearing impairment.

## 4 DISCUSSION

The aim of this study was to investigate if the presence of dementia related neuropathology was associated with an increased presence of hearing impairment in later life. Moderate to frequent densities of neuritic plaques were associated with higher odds of hearing impairment ante‐mortem. Presence of any LB disease was likewise associated with hearing impairment, but this did not increase with higher cortical pathology. Nonspecific amyloid deposition, NFT staging, overall AD neuropathology, and cerebrovascular disease were not clearly associated with increased risks of hearing impairment.

**FIGURE 4:**
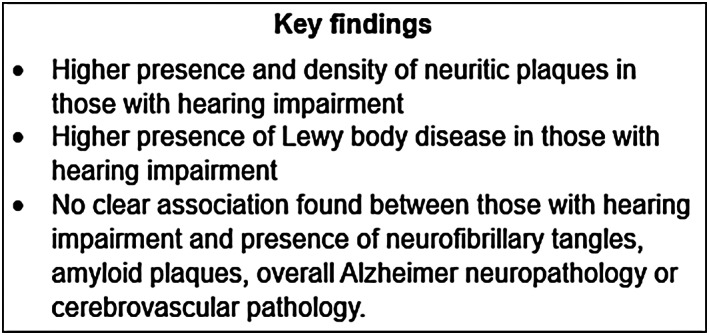
Summary of key findings.

The association of pathologically confirmed neuritic plaques, but not overall amyloid deposition, with hearing impairment fits within broadly inconsistent findings of a relationship between amyloidopathy and hearing impairment in clinical studies; whilst some report associations between amyloid imaging and hearing impairment [21], others report no such association [22]. However, the burden of amyloid pathology alone does not directly correlate with the severity of cognitive impairment [34, 35], and Thal phases of amyloid‐β deposition do not provide any predictive value of clinical outcomes in Alzheimer's disease once neuritic plaques and NFTs have been accounted for [36]. We found neuritic plaques, but not overall amyloid‐β deposition, to be significantly associated with hearing impairment. This is not consistent with the NACC study, which found a negative correlation with neuritic plaques [24]. Of note, CERAD scores provide an estimate of neuritic plaque burden which represent both amyloid‐β and tau (dystrophic neurites) deposition and more reliably predict cognitive impairment [36], hence our finding of an association between CERAD scores and hearing impairment could be a statistical artefact whereby higher CERAD score is a proxy measure for presence of cognitive impairment, with the latter driving the association between CERAD score and hearing impairment. However, in our sensitivity analysis restricted to only those with cognitive impairment, we found that CERAD score was still predictive of greater likelihood of hearing impairment. Additionally, if cognitive impairment were a causative mediator, we would also expect NFT tau pathology to show a clear association with hearing impairment, which was not seen (Figure 4).

The presence of any LB disease was associated with robustly higher odds of hearing impairment being present. However, this was attenuated, rather than strengthened, in higher cortical LB pathology. This could reflect the low number of neocortical LB cases providing less statistical power or suggest that the association between LB pathology and hearing impairment is not secondary to cognitive impairments related to neocortical LB disease. Sensory and bodily symptoms are recognised early manifestations of Lewy body disease often prior to onset of cognitive symptoms, with hyposmia [37], impaired sensory evidence accumulation [38], visual perceptual abnormalities [39], and auditory hallucinations [40] being common. Such ‘sensorial frailty’ [13] manifesting as ARHL may therefore be a characteristic of Lewy body disease even without widespread neocortical pathology and cognitive symptoms. Amygdala LB pathology has been repeatedly linked to the presence of visual hallucinations [31, 40], supporting a possible link to abnormal integration of sensory evidence in lower staged LB pathology. As with neuritic plaque pathology, there may therefore be a direct relationship between early Lewy body pathology and auditory dysfunctions.

Hallmark pathological findings of cerebrovascular disease—infarcts, cerebral amyloid angiopathy, and white matter arteriolosclerosis—were not found to be associated with greater risks of hearing impairment either in isolation or in combination. This is in contrast to previous studies which reported that the presence of white matter hyperintensities (WMH) predicted hearing impairment in older adults [41], and that subclinical atherosclerosis carried a 15% risk of developing ARHL in a 5 year follow up period [42]. However, these pathological changes are not directly assessed within the VCING criteria, reflecting a limitation of the data available.

This study benefits from comprehensive neuropathological assessment, drawing from multiple sites across England and Wales, with several hundred brain tissue donors having provided ante‐mortem clinical information. There are some limitations, however. Hearing impairment was based on judgement of the researcher at the ante‐mortem assessment, incorporating self‐reported information and informant perspectives. These ratings are therefore not objective and may be inaccurate—particularly in those with loss of insight into their symptoms.

Different dementia subtypes have been reported to have distinct auditory dysfunctions—LB with auditory hallucinations, AD with altered speech in noise perception and FTLD with distorted pitch and timbre perception [14]—with WMH specifically correlated with lower frequency hearing rather than high frequencies [41]. Objective measurement of pitch specific audiometry might therefore enrich future research, as such data were not available to us. Investigations utilising both objective and subjective hearing data would be most useful in our attempts to understand the link between ARHL and dementia. Relatedly, whilst there were longitudinal reassessments of cognitive and clinical function, without objective repeat measurement of hearing function we are unable to assess the time course of hearing impairment onset or development in relation to cognitive symptom onset.

Whilst this sample was drawn from a broad geographical area of the United Kingdom, this is not necessarily representative of the older population overall, as looking at Table 1, baseline characteristics of the cohort, − we see that the hearing impaired group are approximately 6 years older at both review and death, have a higher level of education (3.3 years longer) and have less cognitive impairment (51.6% vs. 64.2%) This is unusual as, as aforementioned, hearing impairment predicts cognitive impairment [4, 7] and neurodegeneration [5]. Individuals from the least deprived areas of the United Kingdom are usually over‐represented in UK brain banks [43] and we know socioeconomic deprivation is associated with higher levels of hearing impairment in working‐age [44] and lower levels of hearing aid adoption throughout life [45]. So it seems our hearing impaired group is perhaps, coincidentally, of higher socioeconomic status and health than expected. Additionally, 99.1% self‐reported as ethnically White British. Cohort studies in the USA have found that ARHL is more common in older white adults [46]. Therefore, with the homogeneity of our cohort, we are unable to assess the extent to which social or ethnic inequalities might contribute to ARHL in different pathologies.

## 5 CONCLUSIONS

Whilst neuritic plaques were robustly associated with hearing impairment, overall amyloid deposition, NFTs (and correspondingly, overall AD pathology level) were not, though with consistently positive but uncertain and consequently non‐significant effects. This inconsistency between pathological measures in both this work, and the wider literature, may point to a lack of robustness in the observed associations, or reflect differences in the pathological grading systems: CERAD scores measure the density of neuritic plaques semi‐quantitatively, whereas Braak staging measures the regional spread of pathology but not the regional burden. Semi‐quantitative rating of tau burden, rather than spread, may clarify whether this association is consistent across Alzheimer's disease related pathological changes. However, some pathologies, particularly amyloid‐β and tau [47], play a synergistic role, which may explain this uncertainty. Future research may therefore examine the possible cumulative effect of multiple pathologies on hearing impairment.

The association between dementia and hearing loss is likely multifactorial but our findings suggest ARHL may be a factor predicting increased risk for Lewy Body dementia and Alzheimer's dementia, but not for cerebrovascular dementia. The strength of such a relationship and thus its value clinically needs further investigation including objective measures of hearing impairment. Continued efforts with objective and subjective hearing data and large, heterogeneous cohorts with both ante‐mortem and post‐mortem data are needed to further elucidate the association between ARHL and dementia, hopefully leading to improved accuracy of dementia subclassification.

## AUTHOR CONTRIBUTIONS

**Jessica A. Katanga:** Conceptualisation, Methodology, Software, Investigation, Writing, Review and Editing. **Calum A. Hamilton:** Methodology, Software, Validation, Review and Editing. **Lauren Walker:** Resources, Review and Editing. **Johannes Attems:** Resources, Review and Editing. **Alan J. Thomas:** Conceptualisation, Review and Editing, Funding Acquisition, Project Administration, Supervision.

## FUNDING STATEMENT

This research was funded by NIHR Newcastle Biomedical Research Centre (BRC) and BDR, which is jointly funded by Alzheimer's Research UK and the Alzheimer's Society.

## CONFLICT OF INTEREST STATEMENT

Alan Thomas is Director of the Brains for Dementia Research programme. All other authors have no competing interests.

## ETHICS STATEMENT

Ethical approval for this study was given by NHS Health Research Authority North East—Newcastle & North Tyneside 1 Research Ethics Committee (18/NE/0124).

## Data Availability

The data that support the findings of this study are available from UK Brain Bank Network and Dementias Platform UK and are available upon request.
